# Variational Hybrid Monte Carlo for Efficient Multi-Modal Data Sampling

**DOI:** 10.3390/e25040560

**Published:** 2023-03-24

**Authors:** Shiliang Sun, Jing Zhao, Minghao Gu, Shanhu Wang

**Affiliations:** School of Computer Science and Technology, East China Normal University, Shanghai 200062, China; slsun@cs.ecnu.edu.cn (S.S.); guminghao1081@gmail.com (M.G.); wsh18896656432@163.com (S.W.)

**Keywords:** Markov chain Monte Carlo, Hamiltonian Monte Carlo, Langevin dynamics, multi-modal sampling, variational distribution

## Abstract

The Hamiltonian Monte Carlo (HMC) sampling algorithm exploits Hamiltonian dynamics to construct efficient Markov Chain Monte Carlo (MCMC), which has become increasingly popular in machine learning and statistics. Since HMC uses the gradient information of the target distribution, it can explore the state space much more efficiently than random-walk proposals, but may suffer from high autocorrelation. In this paper, we propose Langevin Hamiltonian Monte Carlo (LHMC) to reduce the autocorrelation of the samples. Probabilistic inference involving multi-modal distributions is very difficult for dynamics-based MCMC samplers, which is easily trapped in the mode far away from other modes. To tackle this issue, we further propose a variational hybrid Monte Carlo (VHMC) which uses a variational distribution to explore the phase space and find new modes, and it is capable of sampling from multi-modal distributions effectively. A formal proof is provided that shows that the proposed method can converge to target distributions. Both synthetic and real datasets are used to evaluate its properties and performance. The experimental results verify the theory and show superior performance in multi-modal sampling.

## 1. Introduction

Generating samples from many distributions encountered in Bayesian inference and machine learning is difficult. Markov Chain Monte Carlo (MCMC) provides a robust framework to generate samples from complex target distributions. Through constructing specific Markov chains, the MCMC methods can converge to the correct target distribution with the chains evolving. Presently, MCMC plays an essential role in artificial intelligence applications and probability inference, especially for estimating the expectations of the target functions [1].

Sampling methods based on dynamics are one of the most popular MCMC methods. The most commonly used dynamics in MCMC are Langevin dynamics and Hamiltonian dynamics. Hamiltonian Monte Carlo (HMC) [2,3] has become one of the most popular MCMC algorithms in Bayesian inference and machine learning. Unlike previous MCMC algorithms [4], HMC takes advantage of the gradient information to explore the continuous probability density function (PDF), which makes HMC more efficient to converge to the target distribution. In particular, HMC transforms the PDF into the potential energy function and adds the kinetic energy function to simulate the motion of the particle in the particular phase space, and thus HMC can satisfy the ergodic property. In practice, HMC exploits the Hamiltonian equation to calculate the new state of the proposed points in the phase space. To maintain a detailed balance, the Metropolis–Hasting (MH) technique is adopted [5]. Gradient information helps to discover and explore the phase space more efficiently, and there is much further research on how to better leverage gradients for HMC [6,7].

Nevertheless, samplers based on dynamics still have some deficiencies. The traditional dynamics samplers [8] and their developments [7,9,10,11,12] have excellent performance in unimodal distributions. However, when facing multi-modal distributions, these algorithms may reveal some problems, especially when the modes are far away from each other. When the modes are close to each other, the momentum variable in dynamics samplers may offer chances for the sample to jump into different modes. When the modes are isolated, the momentum variable cannot jump out of the current mode, because the interval between the two modes has tremendous potential energy. Generally, objects tend to stay in low-energy places which are also referred as high-probability regions. Although we can enlarge the momentum variable to pass through high-potential-energy places, the momentum variable should be exponential-order large, which causes a rapid decrease in the performance of the samplers. To deal with the problem of multi-modal sampling, several studies have been developed [13,14,15,16]. The authors in [13] introduced temperature in the simulation, and simulated annealing is used to gradually reduce the temperature from a high initial value to the value at which we wish to sample. Parallel tempering [17] is one variant of the simulated annealing, which performs inter-distribution exchange with multiple Markov chains to make the sampler explore the state space more freely, without changing the resulting sample distribution. The authors in Sminchisescu and Welling [14] proposed a new dynamics sampler which is based on a darting algorithm [18]. Umbrella sampling [19] is another precise sampling method, which divides the reaction coordinate space into several windows with bias potential to calculate the unbiased free energy in each window. However, when the dimensions are high, these algorithms may have elaborate set-ups and large computational complexity, resulting in low efficiency. The authors in Lan et al. [15] used the natural gradient of the target distribution to establish paths between different modes, and thus samples can jump through the low-probability regions. This method may suffer from low effective sample size (ESS) [3], which means that the independence between two neighbor samples is low. The authors in Tripuraneni et al. [16] introduced the concept of magnetic fields. By means of constructing a dynamics system based on a magnetic field, this method can achieve great performance in multi-modal sampling. However, the setting of the magnetic field parameter is difficult, and this method may also suffer from low ESS in multi-modal sampling problems.

In this paper, we introduce a novel dynamics MCMC method called the variational hybrid Monte Carlo (VHMC). We first improve Hamiltonian dynamics through Langevin dynamics to reduce the autocorrelation of samples and accelerate the convergence of the dynamics sampler. Furthermore, we exploit the variational distribution [20] of the target distribution to help the dynamics sampler to find the new mode. A new Metropolis–Hasting criterion is proposed to satisfy the detailed balance condition [5]. We find that VHMC can overcome the distant multi-modal sampling problem since dynamics-based samplers can sample unimodal distributions well and variational distribution guides the dynamics-based sampler to jump between different modes. Finally, detailed proof is given to demonstrate that our algorithm can converge to the target distribution.

Both synthetic data and real data experiments are conducted to verify our theory. We sample points from seven different Gaussian mixture distributions whose dimensions range from 2 to 256. We apply our method to two-class classification exploiting Bayesian logistic regression [21] to test the performance of VHMC on real datasets. Evaluation indices such as maximum mean discrepancy [22] and autocorrelation are calculated to assess the quality of samples. Experiment results illustrate that the proposed method is capable of sampling from distant multi-modal distribution while obtaining better performance compared with other state-of-the-art methods [16,23].

The main contributions of this work can be summarized as follows. We propose a novel sampler called Langevin Hamiltonian Monte Carlo (LHMC), which achieves lower autocorrelation and faster convergence compared with the HMC sampler. Since we introduce random factors in LHMC, the total energy of the system changes during the simulation, so we design a new Metropolis–Hasting procedure to keep the detailed balance. In addition, to improve the poor performance of the LHMC sampler in multi-modal sampling, we propose a new method VHMC, which uses the variational distribution of the target distribution to guide the sampler to jump through different modes. We use Adam [24] to find the local points with the highest probability density value, and we use these points to construct a mixture of Gaussian as the variational distribution of the target distribution. Detailed proof is given to prove the correctness of our method.

The rest of this article is organized as follows. In Section 2, we review the preliminaries of our study, including Hamiltonian Monte Carlo and Langevin dynamics. Then, we introduce our LHMC sampler and show the objective function in Section 3. In Section 4, we propose the variational hybrid Monte Carlo, which aims to address the problem of multi-modal sampling. Experiments are reported in Section 5. Discussion and conclusions are summarized in Section 6 and Section 7.

## 2. Preliminaries

In this section, we introduce the basic methods exploited in our method. First, we introduce Hamiltonian Monte Carlo, a widely used MCMC sampler in Bayesian machine learning. Then, Langevin dynamics is introduced to improve the performance of the HMC sampler.

### 2.1. Hamiltonian Monte Carlo

Hamiltonian Monte Carlo (HMC) [2,3] is one of the state-of-the-art Markov chain Monte Carlo algorithms. The use of gradient information of the target distribution makes HMC more efficient than the traditional Metropolis–Hasting (MH) algorithms, which employ the random-walk scheme to explore the state space.

HMC exploits Hamiltonian dynamics to calculate the new state, whose state space is composed of joint Gaussian momentum *p* and position θ, where variable *p* is independent with variable θ. Suppose we use a spring oscillator to describe the Hamiltonian dynamics. Then, we can obtain the following equation:

(1) H(θ,p)=U(θ)+K(p),

where U(θ) represents the potential energy of the ball at position θ at time *t* while K(p) represents the kinetic energy of the ball at time *t*. H(p,θ) represents the total energy of the ball. To construct Hamiltonian dynamics, the derivatives of position θ and momentum *p* about time are required. The Hamiltonian equations are formed as follows:

(2) $$\begin{array}{cc} \frac{d\theta }{dt} & =\frac{\partial H(p,\theta )}{\partial p}=\nabla_{p}K\left(p\right)\\ \frac{dp}{dt} & =-\frac{\partial H(p,\theta )}{\partial \theta }=-\nabla_{\theta }U\left(\theta \right). \end{array}$$

In practice, it is difficult to obtain the exact solutions to these differential equations. HMC instead discretizes these equations by using non-zero time steps, which inevitably introduces some error. It is, nevertheless, necessary to use a discretization for which Liouville’s theorem holds exactly [8]. The common discretization method of HMC is leapfrog, which takes the following form:

(3) $$\begin{array}{cc} p_{t+\frac{\epsilon }{2}} & =p_{t}-\frac{\epsilon }{2}\nabla_{\theta }U\left(\theta_{t}\right)\\ \theta_{t+\epsilon } & =\theta_{t}+\epsilon \nabla_{p}K\left(p_{t+\frac{\epsilon }{2}}\right)\\ p_{t+\epsilon } & =p_{t+\frac{\epsilon }{2}}-\frac{\epsilon }{2}\nabla_{\theta }U\left(\theta_{t+\epsilon }\right), \end{array}$$

where ϵ represents the step size. Leapfrog preserves the phase space volume and is also time-reversible. Through the discretization method, we can obtain the new state, and HMC methods then apply Metropolis–Hasting to the new state to decide whether to accept or reject the state, which takes the following form:

(4) $$\begin{array}{c} \min\left[1,\exp\left(H\left(\theta_{n-1},p_{n-1}\right)-H\left(\hat{\theta },\hat{p}\right)\right)\right], \end{array}$$

where $\left(\theta_{\left(n-1\right)},p_{\left(n-1\right)}\right)$ represents the last state and $\left(\hat{\theta },\hat{p}\right)$ represents the newly proposed state. By means of controlling the leapfrog size *L* and small step ϵ, we can adjust the acceptance rate of the HMC sampler. Algorithm 1 gives the pseudo-code of HMC [3].

Suppose we need to sample from the distribution of θ given the observation data D:

(5) $$p\left(\theta |D\right)\propto e^{-U(\theta )},$$

where we have the form of potential energy:

(6) U(θ)∝−lnp(θ|D).

**Algorithm 1:** Hamiltonian Monte Carlo [2]**Input:** Step size ϵ, leapfrog size *L*, starting point $\theta^{\left(1\right)}$, sample number *N***Output:** Samples $\theta_{\left(1:N\right)}$**for** n=1 **to** *N* **do**    $p_{\left(n\right)}∼N\left(0,1\right)$    $\left(\theta_{0},p_{0}\right)=\left(\theta_{\left(n\right)},p_{\left(n\right)}\right)$    $p_{0}=p_{0}-\frac{\epsilon }{2}\nabla_{\theta }U\left(\theta_{0}\right)$    $\theta_{0}=\theta_{0}+\epsilon \nabla_{p}K\left(p_{0}\right)$    **for** i=1 **to** *L* **do**        $p_{i}=p_{i-1}-\epsilon \nabla_{\theta }U\left(\theta_{i-1}\right)$        $\theta_{i}=\theta_{i-1}+\epsilon \nabla_{p}K\left(p_{i}\right)$    **end for**    $p_{L}=p_{L}-\frac{\epsilon }{2}\nabla_{\theta }U\left(\theta_{L}\right)$    $\left(\hat{\theta },\hat{p}\right)=\left(\theta_{L},p_{L}\right)$    u∼Uniform(0,1)    $\alpha =\min\left[1,\exp\left(\left(U\left(\theta_{\left(n\right)}\right)+K\left(p_{\left(n\right)}\right)\right)-\left(U\left(\hat{\theta }\right)+K\left(\hat{p}\right)\right)\right)\right]$    **if** α>u **then**        $\left(\theta_{\left(n+1\right)},p_{\left(n+1\right)}\right)=\left(\hat{\theta },\hat{p}\right)$    **else**        $\left(\theta_{\left(n+1\right)},p_{\left(n+1\right)}\right)=\left(\theta_{\left(n\right)},p_{\left(n\right)}\right)$    **end if****end for**

    According to the Hamiltonian dynamics, through introducing a set of auxiliary momentum variables *p*, HMC sampler can sample the joint distribution π(θ,p) defined as:

(7) $$\pi \left(\theta ,p\right)\propto e^{-H(\theta ,p)}.$$

By using the leapfrog Equation (3), we obtain the new state of θ and *p*. Because the position variable θ and the momentum variable *p* are independent, sampling θ and *p* alternatively will not affect the results.

The Hamiltonian dynamics have three properties. First, it preserves total energy $H\left(\theta_{\left(t\right)},p_{\left(t\right)}\right)=H\left(\theta_{\left(0\right)},p_{\left(0\right)}\right)$, and thus the joint probability density has $p\left(\theta_{\left(t\right)},p_{\left(t\right)}\right)=p\left(\theta_{\left(0\right)},p_{\left(0\right)}\right)$. Second, it satisfies volume conservation. Last, it is time-reversible [25]. As a result, if the potential energy and the kinetic energy remain unchanged during the dynamics system, then the joint probability density of θ and *p* also remains unchanged.

Compared with the random-walk strategy, HMC methods explore the target distribution much more efficiently due to the use of gradient information. HMC can travel a long distance in the phase space (θ,p), which enhances the acceptance rate. However, it is really difficult for HMC to travel across the low-probability region, in which the value of the gradient of the potential energy is very large. Enlarging the momentum variable *p* may be helpful to jump over these regions, but the efficiency of HMC may decrease significantly.

Recently, some new developments of HMC have been proposed to make the HMC sampler more flexible. For example, Riemann manifold HMC [12] exploits the Riemann geometry to tune the mass *M*, which tends to create a more efficient HMC sampler. The “No U-Turn” sampler [7] can tune the step size ϵ, leapfrog length *L* and the simulation steps automatically. We note that, in principle, these state-of-the-art HMC samplers can also be combined with our proposed method.

### 2.2. Langevin Dynamics

Langevin dynamics were first used to describe the diffusion process of molecular systems, which was then integrated into MCMC samplers [26,27,28,29]. Langevin dynamics are a system of Ito-type stochastic differential equations, which take the following form:

(8) $$\begin{array}{c} d\theta =-M^{-1}\nabla U\left(\theta \right)dt+\sqrt{\frac{2\beta^{-1}}{M}}dW, \end{array}$$

where *W* represents the stochastic Wiener process, and *M* represents the diagonal mass matrix. U(θ) represents the energy function and $\beta^{-1}=k_{B}T$, where $k_{B}$ is Boltzmann constant and *T* represents the temperature. Since solving (8) is difficult, the Euler–Maruyama discretization [30] is used to approximately solve the differential equation, which takes the following form:

(9) $$\begin{array}{c} \theta_{n+1}=\theta_{n}-\frac{\epsilon^{2}}{2}M^{-1}\nabla U\left(\theta_{n}\right)+\sigma \epsilon^{2}\sqrt{\frac{2\beta^{-1}}{M}}z_{n}, \end{array}$$

where z∼N(z|0,I), and ϵ represents the integration step size. Please note that (9) only gives the solution to the overdamped Langevin dynamics [31], which means that the friction term has not been concerned. The Langevin dynamics with the friction term [32] is defined as:

(10) $$\begin{array}{cc} d\theta & =M^{-1}pdt\\ dp & =-\nabla U\left(\theta \right)dt-\gamma pdt+\sigma \sqrt{M}dW, \end{array}$$

where γ>0 represents the friction factor and $\sigma =\sqrt{2\gamma \beta^{-1}}$. However, simulating (10) is very difficult, and thus a discretization method [31] was used to solve the above stochastic differential equation, which takes the following form:

(11) $$\begin{array}{cc} p_{(n+1)/2} & =p_{n}-\frac{\epsilon }{2}\nabla U\left(\theta_{n}\right)\\ \theta_{(n+1)/2} & =\theta_{n}+\frac{\epsilon }{2}M^{-1}p_{(n+1)/2}\\ {\hat{p}}_{(n+1)/2} & =a_{1}p_{(n+1)/2}+a_{2}\sqrt{M}z_{n+1}\\ \theta_{n+1} & =\theta_{(n+1)/2}+\frac{\epsilon }{2}M^{-1}{\hat{p}}_{(n+1)/2}\\ p_{n+1} & ={\hat{p}}_{(n+1)/2}-\frac{\epsilon }{2}\nabla U\left(\theta_{n+1}\right), \end{array}$$

where z∼N(z|0,I), ϵ represents the step size and $a_{1}=e^{-\gamma \epsilon }$ and $a_{2}=\sqrt{\beta^{-1}\left(1-a_{1}^{2}\right)}$.

Compared with Hamiltonian dynamics, Langevin dynamics allow the exploration of the state space more freely, for Langevin dynamics concern more random factors. Please note that in (9), Langevin dynamics added Brownian movement, which provides more flexibility for exploring the state space.

## 3. Langevin Hamiltonian Monte Carlo

HMC exploits the Hamiltonian dynamics to propose the new sample. However, the HMC sampler may have large autocorrelation in that each new sample is obtained from the deterministic calculation of the last sample. Specifically, Equation (3) defines the process of calculating a new state $\left(\theta_{t+1},p_{t+1}\right)$ through the old state $\left(\theta_{t},p_{t}\right)$. HMC can reduce autocorrelation by increasing the value of the leapfrog size. However, it will make the sampler inefficient.

To further improve the performance of the autocorrelation of the HMC sampler, we propose the Langevin Hamiltonian Monte Carlo (LHMC). The main idea of LHMC is to take advantage of Langevin dynamics to add randomness to the proposed state. In Langevin dynamics, we consider that the total energy consists of the potential energy, kinetic energy and internal energy, which takes the following:

(12) $$\begin{array}{c} H=U(\theta )+K(p)+Q, \end{array}$$

where *Q* represents the internal energy. The random thermal motion consumes the internal energy which finally transforms into the kinetic energy, which is described as:

(13) $$\begin{array}{cc} p_{t} & =a_{1}p_{t-1}+a_{2}\sqrt{M}z\\ \Delta E & =K\left(p_{t}\right)-K\left(p_{t-1}\right)\\ Q_{t} & =Q_{t-1}-\Delta E. \end{array}$$

We use the Metropolis–Hasting criterion to accept the samples, so the acceptance rate φ takes the following form:

(14) $$\begin{array}{cc} \varphi & =min\left(1,\exp\left(\left(U_{t-1}+K_{t-1}+Q_{t-1}\right)-\left(U_{t}+K_{t}+Q_{t}\right)\right)\right)\\ \; & =min\left(1,\exp\left(\left(U_{t-1}+K_{t-1}+Q_{t-1}\right)-\left(U_{t}+K_{t}+Q_{t-1}-\Delta E\right)\right)\right)\\ \; & =min\left(1,\exp\left(\left(U_{t-1}+K_{t-1}\right)-\left(U_{t}+K_{t}-\Delta E\right)\right)\right). \end{array}$$

The detailed algorithms of LHMC are illustrated in Algorithm 2. Given the target distribution, LHMC exploits Langevin dynamics and Hamiltonian dynamics to explore state space via the discretization of LHMC, which can be summarized as three substages shown in Algorithm 3. The first substage is Langevin dynamics, which takes form as (15)–(17). The second substage is Hamiltonian dynamics, which takes form as (3), and the last substage is also Langevin dynamics. Assume the initial state is (θ,p); then, a half update of the Langevin dynamics can be written as:

(15) $$\begin{array}{cc} p_{(n+1)/2} & =p_{n}-\frac{\epsilon }{2}\nabla U\left(\theta_{n}\right)\\ \theta_{(n+1)/2} & =\theta_{n}+\frac{\epsilon }{2}M^{-1}p_{(n+1)/2}. \end{array}$$

The random thermal motion of molecules takes the following form:

(16) $$\begin{array}{cc} {\hat{p}}_{(n+1)/2} & =a_{1}p_{(n+1)/2}+a_{2}\sqrt{M}z_{n+1}. \end{array}$$

The other half update of the Langevin dynamics can be written as:

(17) $$\begin{array}{cc} \theta_{n+1} & =\theta_{(n+1)/2}+\frac{\epsilon }{2}M^{-1}\\ p_{n+1} & ={\hat{p}}_{(n+1)/2}-\frac{\epsilon }{2}\nabla U\left(\theta_{n+1}\right). \end{array}$$

**Algorithm 2:** Langevin Hamiltonian Monte Carlo (LHMC)**Input:** Step size ϵ, leapfrog size *L*, starting point $\theta_{\left(1\right)}$, sample number *N***Output:** Samples $\theta_{\left(1:N\right)}$**for** n=1 **to** *N***do**     $\hat{\theta },\hat{p},\Delta E=\mathrm{DLHMC}\left(\theta_{n},p,\epsilon ,L\right)$ //(      # see in Algorithm 3(     u∼Uniform(0,1)     $\alpha =\min\left(1,\exp\left(U\left(\theta_{\left(n-1\right)}+K\left(p_{\left(n-1\right)}\right)\right)-\left(U\left(\hat{\theta }\right)+K\left(\hat{p}\right)-\Delta E\right)\right)\right)$     **if** α>u **then**         $\left(\theta_{\left(n+1\right)},p_{\left(n+1\right)}\right)=\left(\hat{\theta },\hat{p}\right)$    **else**         $\left(\theta_{\left(n+1\right)},p_{\left(n+1\right)}\right)=\left(\theta_{\left(n\right)},p_{\left(n\right)}\right)$    **end if****end for**

**Algorithm 3:** Discretization for Langevin Hamiltonian Monte Carlo (DLHMC)
**Input:** Step size ϵ, leapfrog size *L*, starting point $\left(\theta_{\left(n\right)},p_{\left(n\right)}\right)$**Output:** $\theta_{\left(n+1\right)}$, $p_{\left(n+1\right)}$, ΔE

ΔE=0

Obtaining $\theta_{(n+1)/6},p_{(n+1)/6}$ through (15).

$\hat{p}=p_{(n+1)/6}$

Obtaining the thermal motion of molecules $p_{(n+1)/6}$ through (16).

$\Delta E=\Delta E+K\left(p_{(n+1)/6}\right)-K\left(\hat{p}\right)$

Obtaining $\theta_{(n+1)/3},p_{(n+1)/3}$ through (17).Obtaining $\theta_{2(n+1)/3},p_{2(n+1)/3}$ by simulating Hamiltonian dynamics through (3).Obtaining $\theta_{5(n+1)/6},p_{5(n+1)/6}$ through (15).

$\hat{p}=p_{5(n+1)/6}$

Obtaining the thermal motion of molecules $p_{5(n+1)/6}$ through (16).

$\Delta E=\Delta E+K(p_{5(n+1)/6)}-K\left(\hat{p}\right)$

Obtaining $\theta_{\left(n+1\right)},p_{\left(n+1\right)}$ through (17).


**Figure 1:**
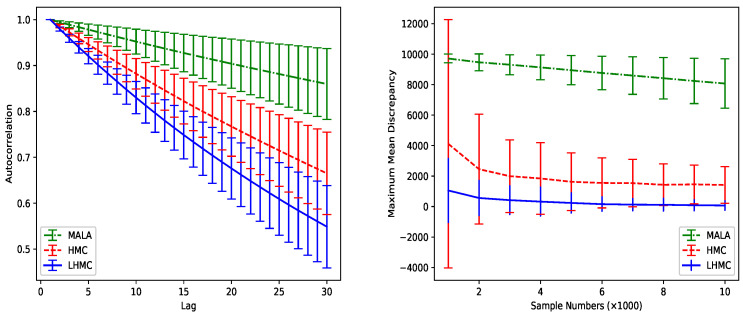
The comparison of autocorrelation and maximum mean discrepancy (MMD) for three different methods. The (**left**) column shows the relationship between autocorrelation and lag of sample number after the burn-in period. The (**right**) column demonstrates the relationship between MMD and sample number (best viewed in color).

We demonstrate the performance of LHMC on a strongly correlated Gaussian with variances $\left[10^{2},10^{-2}\right]$ rotated by $\frac{\pi }{4}$, which is an extreme circumstance of Brooks et al. [3]. Each method in Figure 1 is run for 10,000 iterations with 1000 burn-in samples. We experimented 100 times and calculated the mean and variance of autocorrelation and maximum mean discrepancy. We set M=1.2I where I is an identity matrix with the same dimension as the sample distribution, γ=0.5, T=200, ϵ=0.05, leapfrog size L=40. As Figure 1 illustrates, LHMC achieves lower autocorrelation and faster convergence rate compared with HMC and Metropolis-adjusted Langevin algorithm (MALA) [29].

## 4. Variational Hybrid Monte Carlo

LHMC method, as a dynamics-based MCMC sampler, cannot sample from multi-modal distributions well when the modes are far away from each other, especially in high dimensions [15,16]. In this section, we present a novel MCMC method called variational hybrid Monte Carlo (VHMC) to tackle the above issue and prove that it can target the correct distribution.

### 4.1. Variational Hybrid Monte Carlo

Recent studies [15,16] have shown that MCMC samplers based on dynamics are challenging when dealing with multi-modal distributions, since there exist low-probability regions between the isolated modes. Once the initial point is chosen, these methods could only sample from one mode closer to the initial point.

**Figure 2:**
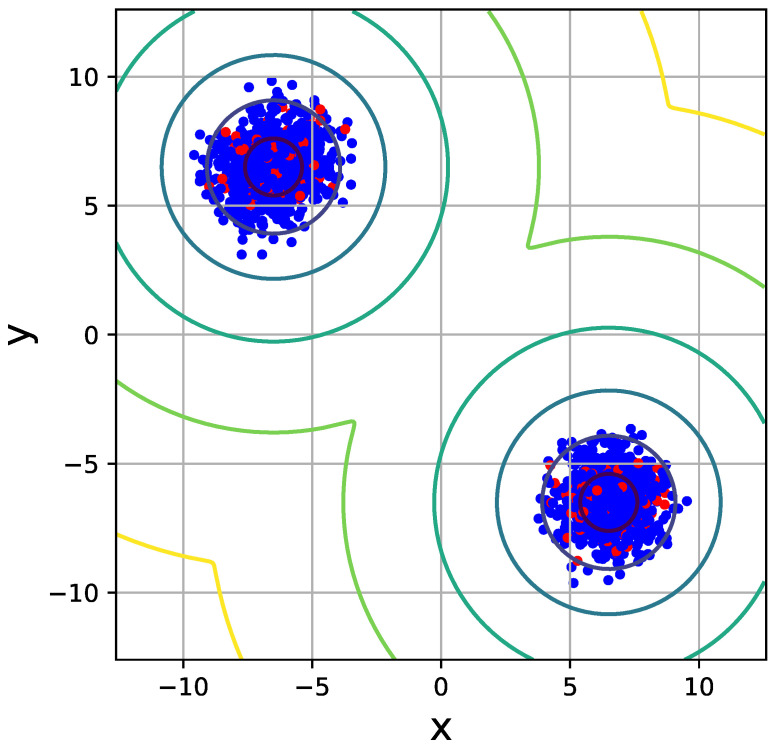
The guide points (red points) when using VHMC (best viewed in color).

In this study, we are aiming to address the problem of multi-modal sampling. We propose a new concept referred to as guide points which are illustrated in Figure 2. Guide points are samples generated from the variational distribution. With the help of these guide points, VHMC can travel across the low-probability regions between two modes. The specific construction of the variational distribution is described as follows.

Suppose we want to sample from the distribution p(θ). To obtain the local points with the highest probability density value, *K* samples are generated from the solution space, and we then use these *K* initial points with Adam [24] to calculate the solutions which take the following form:

(18) $$\begin{array}{cc} g_{t} & =\nabla_{\theta_{k}}\pi \left(\theta_{k(t-1)}\right)\\ m_{t} & =\beta_{1}\cdot m_{t-1}+\left(1-\beta_{1}\right)\cdot g_{t}\\ v_{t} & =\beta_{2}\cdot v_{t-1}+\left(1-\beta_{2}\right)\cdot g_{t}^{2}\\ {\hat{m}}_{t} & =\frac{m_{t}}{{\left(1-\beta_{1}\right)}^{t}}\\ {\hat{v}}_{t} & =\frac{v_{t}}{{\left(1-\beta_{2}\right)}^{t}}\\ \theta_{k(t)} & =\theta_{k(t-1)}-\alpha \cdot \frac{{\hat{m}}_{t}}{\sqrt{{\hat{v}}_{t}}+\phi }, \end{array}$$

where $\theta_{k(t)}$ represents the solution of the *k*-th mode in the *t*-th iteration, π represents the target distribution, α represents the step size and $\beta_{1},\beta_{2}$ represent the exponential decay rates for the moment estimates. From these local points with the highest probability density value, we can obtain *K* modes. For each mode, we use these local points with the highest probability density value as the initial state of LHMC sampler to generate *S* samples. After that, we can obtain the sample sets $mode_{k}=\left\{\theta |\theta ∼p_{k}\left(\theta \right)\right\},k=1,\ldots ,K$, which are generated in the single mode distribution $p_{k}\left(\theta \right),k=1,\ldots ,K$. We assume that each mode follows Gaussian distribution $q_{k}\left(\theta \right),k=1,\ldots ,K$. To use $q_{k}\left(\theta \right)$ to approximate $p_{k}\left(\theta \right)$, we use the KL divergence to quantify the similarity of the two distributions:

(19) $$\begin{array}{cc} \mathrm{KL}(p_{k}\left(\theta \right)||q_{k}\left(\theta \right)) & =\int p_{k}\left(\theta \right)\ln\frac{p_{k}\left(\theta \right)}{q_{k}\left(\theta ;\mu ,\Sigma \right)}d\theta =E_{p_{k}\left(\theta \right)}\left[\ln\frac{p_{k}\left(\theta \right)}{q_{k}\left(\theta ;\mu ,\Sigma \right)}\right], \end{array}$$

(20) $$\begin{array}{c} \min\left(\mathrm{KL}\left(p_{k}\left(\theta \right)||q_{k}\left(\theta \right)\right)\right)=\min\;\left[-\int p_{k}\left(\theta \right)\ln q_{k}\left(\theta ;\mu ,\Sigma \right)d\theta \right]=\max\;\left[E_{p_{k}\left(\theta \right)}\left(\ln q_{k}\left(\theta ;\mu ,\Sigma \right)\right)\right]. \end{array}$$

Since minimizing the integrator in (19) is equivalent to maximizing the likelihood, we translate the problem into solving the likelihood of the Gaussian mixture distribution, which takes the following form:

(21) $$\begin{array}{c} q\left(\theta \right)=\frac{1}{K}\sum_{k=1}^{K}N\left(\mu_{k},\Sigma_{k}\right), \end{array}$$

where $\mu_{k}$ and $\sigma_{k}$ represent the mean and variance of k-th mode, respectively. The whole process of generating the variational distribution (GVD) is demonstrated in Algorithm 4.

Since constructing the variational distribution is to find the modes of the target distribution, this process can be managed parallelly, so the total cost contains two aspects, and they are the cost of Adam and the LHMC algorithm. The effectiveness of Adam makes us find the local optimal solution rapidly. In addition, because the initial samples are given through the Adam algorithm, LHMC can explore the space rapidly without any burn-in samples, which reduces the computational cost.

As shown in Figure 2, we can find that the mean of the variational distribution with guide points is similar to the target distribution, but the variance is different, which demonstrates that our variational method can move the initial random Gaussian to the center of the corresponding mode. If the variance of the initial random Gaussian is too large, the guide points will be scattered without good alignment with the target distribution, which means that the variational distribution loses strong guidance for the sampler trapped in the low-probability regions. Therefore, empirically, we set the random standard Gaussian to initialize the ADAM optimizer to keep a good alignment between guide points and the high probability centers of multiple modes.
**Algorithm 4:** Generating the variational distribution (GVD)**Input:** Population size *K*, number of samples for each population *S*, the target distribution π, step size α, exponential decay rates for the moment estimates $\beta_{1},\beta_{2}$, convergence error ϵ, $\phi =10^{-7}$, step size γ, leapfrog size *L***Output:** The variational distribution *q* of π$\theta_{k}∼N\left(0,I\right),k\in \left[1,K\right]$**for** k=1 **to** *K* **do**     $m_{0},v_{0},t=0$    **while** $\theta_{k(t)}-\theta_{k(t-1)}>\epsilon$ **do**         t=t+1         $g_{t}=\nabla_{\theta_{k}}\pi \left(\theta_{k(t-1)}\right)$         $m_{t}=\beta_{1}\cdot m_{t-1}+\left(1-\beta_{1}\right)\cdot g_{t}$         $v_{t}=\beta_{2}\cdot v_{t-1}+\left(1-\beta_{2}\right)\cdot g_{t}^{2}$         ${\hat{m}}_{t}=\frac{m_{t}}{{\left(1-\beta_{1}\right)}^{t}}$         ${\hat{v}}_{t}=\frac{v_{t}}{{\left(1-\beta_{2}\right)}^{t}}$         $\theta_{k(t)}=\theta_{k(t-1)}-\alpha \cdot \frac{{\hat{m}}_{t}}{\sqrt{{\hat{v}}_{t}}+\phi }$    **end while****end for****for** k=1 **to** *K* **do**    $mode_{k}=\mathrm{LHMC}\left(\gamma ,L,\theta_{k(t)},S\right)$//(      # see in Algorithm 2(    Calculating the mean $\mu_{k}$ and variance $\sigma_{k}$ for $mode_{k}$.**end for**$q=\frac{1}{K}\sum_{k=1}^{K}N\left(\mu_{k},\sigma_{k}\right)$

We generate new samples with two strategies. First, a vast number of samples are generated through the LHMC sampler with probability φ. When LHMC rejects the sample with probability 1−φ, we then generate a sample with probability $\frac{\pi (x)}{cq(x)}$ from the variational distribution, whose derivation is demonstrated in Appendix A. We finally accept the samples generated from the variational distribution through the following equation:

(22) $$\begin{array}{c} \min\left[1,\frac{r(x,x_{r})}{1-\varphi }\right], \end{array}$$

where r(.) represents the rejection probability of the current state jumping into other states. The overall algorithm flow of VHMC is given in Algorithm 5 and the theoretical convergence analysis of VHMC is provided to demonstrate that VHMC could converge to the target distribution in Appendix B.

In the LHMC sampler, we found that in the Metropolis–Hasting procedure, the sampler rejects the proposed sample with probability 1−φ, where φ is defined in (14). This is an interesting phenomenon in that if the proposed sample is rejected, the traditional MCMC samplers would put the sample back to the last position. In our study, if one sample is rejected in the MH step, we generate a new sample from q(θ). We accept the samples generated from the variational distribution through the newly proposed acceptance rate, and thus the detailed balance holds.
**Algorithm 5:** Variational Hybrid Monte Carlo (VHMC)**Input:** Step size ϵ, leapfrog size *L*, starting point $\theta_{\left(1\right)}$, sample number *N*, target distribution π, population size *k*, number of samples for each population *s***Output:** Samples $\theta_{\left(1:N\right)}$q=GVD(k,s,π)//(      # see in Algorithm 4(**for** n=1 **to** *N* **do**        u∼Uniform(0,1)        $\hat{\theta },\hat{p},\Delta E=\mathrm{DLHMC}\left(\theta_{n},p,\epsilon ,L\right)$ //(      # see in Algorithm 3(        u∼Uniform(0,1)        $\varphi =\min\left(1,\exp\left(U\left(\theta_{\left(n\right)}+K\left(p_{\left(n\right)}\right)\right)-\left(U\left(\hat{\theta }\right)+K\left(\hat{p}\right)-\Delta E\right)\right)\right)$        **if** φ>u **then**            $\theta_{\left(n+1\right)}=\hat{\theta }$        **else**            $\theta^{*}∼q\left(\theta \right)$            h∼Uniform(0,1)            **while** $h>\frac{\pi (\theta^{*})}{cq(\theta^{*})}$ **do**               $\theta^{*}∼q\left(\theta \right)$            **end while**            u∼Uniform(0,1)            **if** $u<\min(1,\frac{1-r(\theta^{*},\theta_{r}^{*})}{1-\varphi })$ **then**               $\theta_{\left(n+1\right)}=\theta^{*}$            **else**               $\theta_{\left(n+1\right)}=\theta_{\left(n\right)}$            **end if**     **end if****end for**

### 4.2. Deficiency of Parallel HMC

**Figure 3:**
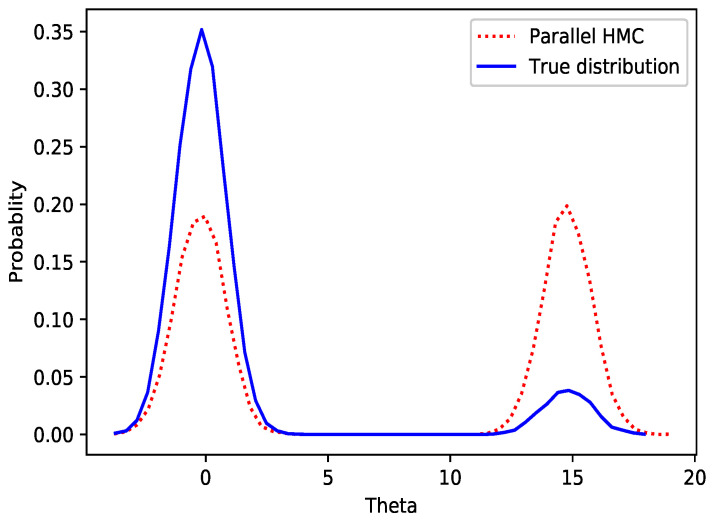
The probability density function between parallel HMC and the actual distribution.

Although we can run *N* HMC samplers in parallel to approximately sample from a multi-modal distribution, in high dimensions, this kind of method is inaccurate. In other words, the probability of each mode may be the same, which cannot reflect the actual distribution. Figure 3 shows that parallel HMC cannot sample from the actual distribution. The problem with parallel HMC is that the gradient direction cannot determine the probability of each mode.

## 5. Experiments

In this section, we investigate the performance of VHMC on multi-modal distributions and real datasets and compare our method with state-of-art algorithms. All our experiments are conducted on a standard computer with a 4.0 GHz Intel core i7 CPU. First, we introduce the performance index that will be used in the following parts.

**Effective sample size**. The variance of a Monte Carlo sampler is determined by its effective sample size (ESS) [3], which is defined as:

(23) $$\mathrm{ESS}=N/(1+2\times \sum_{s=1}^{\infty }\rho \left(s\right)),$$

where *N* represents the number of all the samples and ρ(s) represents the *s*—step autocorrelation where autocorrelation is an index that considers the correlation between two samples. Let *X* be a set of samples, and *t* be the number of iterations (*t* is an integer). Then, $X_{t}$ is the sample at time *t* of *X*. The autocorrelation between time *s* and *t* is defined as:

(24) $$R\left(s,t\right)=\frac{E[\left(X_{t}-\mu_{t}\right)\left(X_{s}-\mu_{s}\right)]}{\sigma_{t}\sigma_{s}},$$

where *E* is the expected value operator. The correlation between two nearby samples can be measured with autocorrelation. The lower the value of autocorrelation, the more independent the samples.

**Maximum mean discrepancy**. The difference between samples drawn from two distributions can be measured as maximum mean discrepancy (MMD) [22], which takes the following form:

(25) $$\begin{array}{cc} MMD^{2}\left[X,Y\right]= & \frac{1}{M^{2}}\sum_{i,j=1}^{M}k\left(x_{i},x_{j}\right)-\frac{2}{MN}\sum_{i,j=1}^{M,N}k\left(x_{i},y_{j}\right)+\frac{1}{N^{2}}\sum_{i,j=1}^{N}k\left(y_{i},y_{j}\right), \end{array}$$

where *M* represents the sample number in *X*, *N* represents the sample number in *Y* and *k* represents the kernel function. By calculating the MMD value, we can analyze the convergence rate of the proposed methods.

**Relative error of mean**. This is a summary of the errors in approximating the expectation of variables across all dimensions [33], which is computed as:

(26) $$REM_{t}=\frac{\sum_{i=1}^{d}|\bar{\theta_{i}^{t}}-\theta_{i}^{*}|}{\sum_{i}|\theta_{i}^{*}|},$$

where $\bar{\theta_{i}^{t}}$ is the average of the *i*’th variable at time *t*, $\theta_{i}^{*}$ is the actual mean value, *d* denotes the dimension of sampling distribution and the denominator $\sum_{i}|\theta_{i}^{*}|$ represents the sum of $|\theta_{i}^{*}|$ on the true distribution.

### 5.1. Mixture of Isotropic Gaussians

We conduct our first experiment on two multi-modal distributions where we consider two simple 2*D* Gaussian mixtures whose distributions are analytically available. First, we consider a Gaussian mixture distribution whose modes are close to each other, and then we consider a Gaussian mixture whose modes are isolated and far away from each other. The distributions are given as follows: $p\left(x\right)=\frac{1}{2}N\left(x;\mu ,\Sigma \right)+\frac{1}{2}\left(x;-\mu ,\Sigma \right)$ for $\sigma_{x}^{2}=\sigma_{y}^{2}=1$, $\rho_{xy}=0$, $x=\left(x,y\right)\in R^{2}$ and μ=(2.5,−2.5) (modes are close to each other) or μ=(6.5,−6.5) (modes are far away from each other). The experiment setting is the same with Tripuraneni et al. [16]. The purpose of the experiments is to sample points that are independently identically distributed in the multi-modal distribution correctly.

In this experiment, we compare MHMC, HMC, MGHMC [23] and parallel tempering (SAMCMC) [17] against VHMC. First, we compare the sample result of these methods intuitively. Then, averaged autocorrelation and MMD are used to compare the performance of each method further. Each method is run for 10,000 iterations with 1000 burn-in samples. The number of leapfrog steps is uniformly drawn from (100−l,100+l) with l=20, which is suggested by Livingstone et al. [34]. We set step size as ϵ=0.05, friction coefficient as γ=0.1 and the initiate position as θ=(0,0). The authors in Tripuraneni et al. [16] indicated that the multi-modal problem is a challenge for HMC samplers. However, we find that HMC samplers can sample points from the multi-modal distribution, especially when the modes are close to each other.

**Figure 4:**
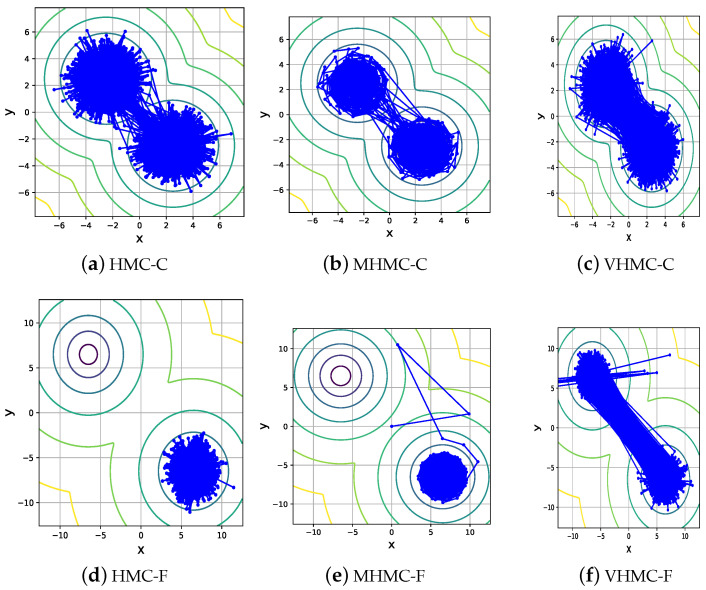
Sampling experiment results for mixture Gaussian distributions. In the first line, HMC-C and MHMC-C and VHMC-C represent HMC, MHMC and VHMC sample from a Gaussian mixture whose modes are close to each other and the mean value of each mode of the Gaussian mixture is $\mu_{0}=\left(2.5,-2.5\right)$ and $\mu_{1}=\left(-2.5,2.5\right)$, respectively. In the second line, HMC-F, MHMC-F and VHMC-F represent HMC, MHMC and VHMC sample from a Gaussian mixture whose modes are far away from each other and the mean value of each mode of the Gaussian mixture is $\mu_{0}=\left(6.5,-6.5\right)$ and $\mu_{1}=\left(-6.5,6.5\right)$, respectively.

Figure 4 clearly shows that when μ=(2.5,−2.5), three methods can sample the multi-modal distribution. Nevertheless, there is some difference between them. MHMC may sample from this mixture on a Gaussian distribution, but it updates state and position only according to random-walk proposals, which hardly jumps to the other sampling mode from a far place. The HMC sampler changes its sampling mode more frequently due to the gradient information of the target distribution. Obviously, combining the help of guide points, Brownian movement and gradient information VHMC changes its mode much more frequently than MHMC and HMC. From the result, we can also conclude that when the modes are close to each other, MHMC and HMC may sample this multi-modal distribution. However, when the modes are isolated and far away from each other with larger μ, for instance, μ=(6.5,−6.5), both MHMC and HMC cannot sample from the target distribution, shown in the second row of Figure 4. This is because only random-walk proposals and gradient information cannot make them directly travel across the large low-probability regions. Nevertheless, our method still performs well by taking advantage of the samples generated from variational distribution to explore the phase space in different modes freely.

For the multiple modes far away from each other, HMC hardly changes its mode, so it converges to the target distribution slowly. On the contrary, VHMC changes the sampling mode very frequently, thus it converges to the target distribution quickly. To compare the convergence rate and the independence of the samples with state-of-the-art sampling methods, we exploit MMD and autocorrelation to describe the performance when sampling the Gaussian mixture.

**Figure 5:**
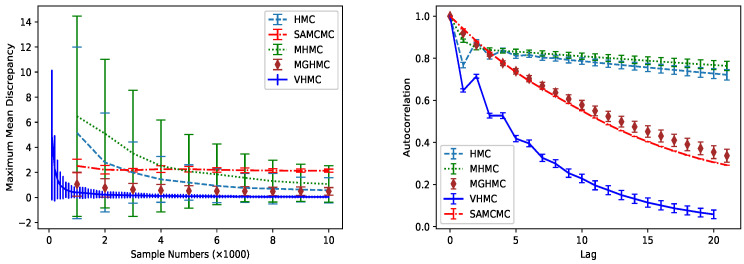
The comparison of MMD and autocorrelation for four different methods. The (**left**) column shows the relationship between MMD and sample number and the (**right**) column demonstrates the relationship between autocorrelation and lag of sample number after the burn-in period.

MMD between exact samples generated from the target density and generated samples is used to describe the convergence performance of the samplers. We use a quadratic kernel [35] $k\left(x,x^{{}^{\prime }}\right)={\left(1+\langle x,x^{{}^{\prime }}\rangle \right)}^{2}$ where 〈·〉 denotes dot product, averaged over 100 runs of the Markov chains. Figure 5 demonstrates that our method achieves the best performance in convergence rate and autocorrelation after the burn-in period. Since our method converges to the target distribution quickly, we furthermore narrow the number of the first 500 samples. In general, comparing Figure 4 and Figure 5, we conclude that the convergence rate is inversely proportional to the MMD and autocorrelation for HMCM, HMC and VHMC.

**Figure 6:**
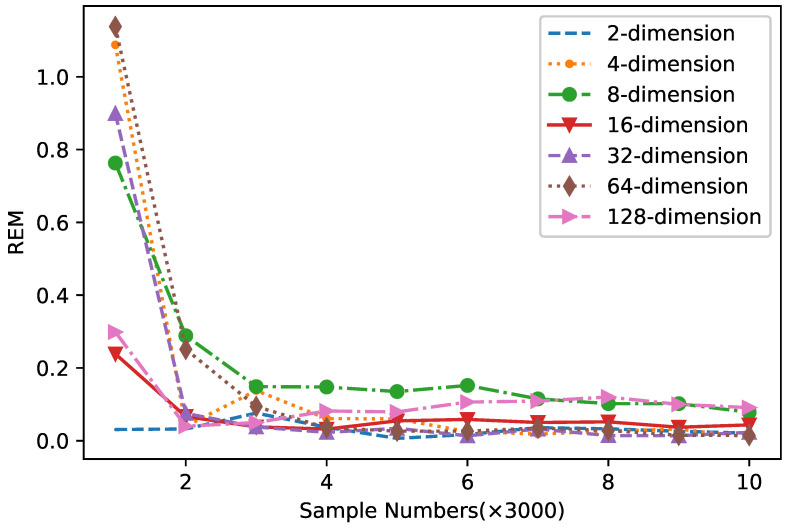
Relative error of mean on high dimensions.

To test the performance of the proposed method on high-dimensional multi-modal distribution, we conduct our experiments on 2 to 128 dimensions. The target distribution is given as:

(27) $$\begin{array}{c} p\left(\theta \right)=\frac{1}{{\left(2\pi \right)}^{\frac{n}{2}}}\left(0.7\cdot \exp\left[\frac{-{\left(x-\mu_{0}\right)}^{⊤}\left(x-\mu_{0}\right)}{2}\right]+0.3\cdot \exp\left[\frac{-{\left(x-\mu_{1}\right)}^{⊤}\left(x-\mu_{1}\right)}{2}\right]\right), \end{array}$$

where $\mu_{0}=\left(a_{1},\ldots ,a_{n}\right),\mu_{1}=\left(b_{1},\ldots b_{n}\right),a_{i}=-1,b_{i}=1$ and *n* equals dimensions. Figure 6 shows that the proposed method has lower REM in high dimensions, which indicates that VHMC can sample from the high-dimensional distant multi-modal distributions.

### 5.2. Mixture of Heterogeneous Gaussians

**Figure 7:**
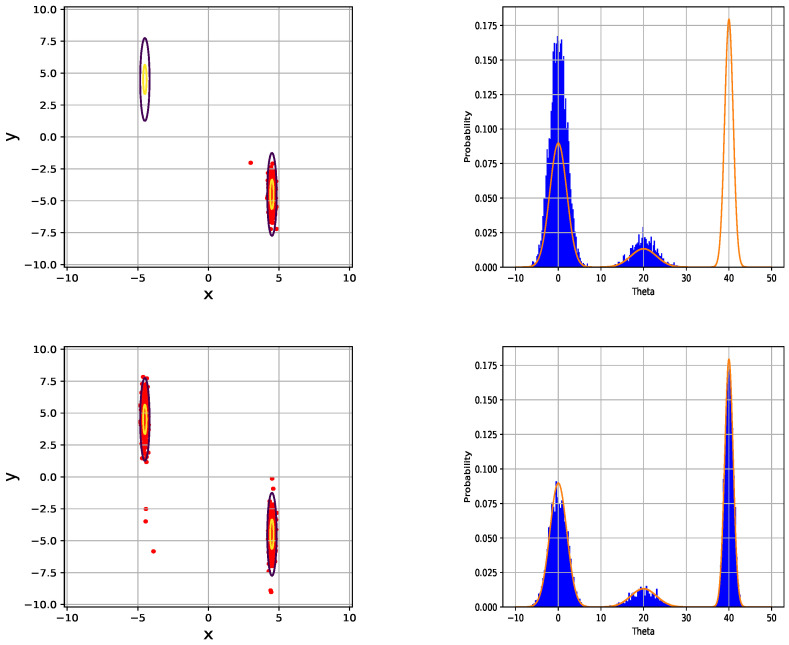
The performance of HMC and VHMC on the mixture of heterogeneous Gaussians. In the first column, we show the scatter diagram of HMC (**upper**) and VHMC (**bottom**). In the second column, we show the histogram of HMC (**upper**) and VHMC (**bottom**).

In the first experiment, we have already discussed the Gaussian mixture when the variance of the modes is the same. In practice, real data distributions often have different variances and probability of modes. To demonstrate strong stability, we construct two new Gaussian mixtures with different variances and probability of modes. The first one is given as follows: $p\left(\theta \right)=\pi_{1}N\left(\theta ;\mu_{1},\sigma_{1}\right)+\pi_{2}N\left(\theta ;\mu_{2},\sigma_{2}\right)+\pi_{3}N\left(\theta ;\mu_{3},\sigma_{3}\right).$ We set $\pi_{1}=0.1$, $\pi_{2}=0.8$, $\pi_{3}=0.1$, $\sigma_{1}=1$, $\sigma_{2}=3$, $\sigma_{3}=2$. The second one takes the following form: $p\left(\theta \right)=0.5N\left(\theta ;\mu_{1},\sigma_{1}\right)+0.5N\left(\theta ;\mu_{2},\sigma_{2}\right)$. Here, we set $\sigma_{x}^{2}=0.01$, $\sigma_{y}^{2}=1$, $\rho_{xy}=0.0$. Similar to the previous experiment, our method runs 10,000 iterations with 1000 burn-in samples. Figure 7 shows that VHMC has strong stability. Even when the variance becomes tiny, our method still shows advanced performance. From the second column of Figure 7, we can also observe that the HMC sampler may sample multi-modal distribution especially when the HMC sampler has chances to jump out of one mode. Although the distance between the left mode and the middle mode is the same as the distance between the middle mode and right mode, the different variances force the HMC sampler to sample from the left two modes.

### 5.3. Bayesian Logistic Regression

Logistic regression (LR) [36] is a traditional method for classification. We optimize the parameters by maximizing the logistic likelihood function. By exploiting the parameters, we can predict the class of the data.

To verify the performance on real datasets, we apply the proposed method to Bayesian logistic regression (BLR) [21] and our method is compared with logistic regression (LR), variational Bayesian logistic regression (VBLR) and HMC.

The likelihood function of a two-class classification problem can be defined as:

(28) $$p\left(t|w\right)=\prod_{n=1}^{N}y_{n}^{t_{n}}{\left[1-y_{n}\right]}^{1-t_{n}},$$

where $t_{n}\in \left\{0,1\right\}$ and $t={\left(t_{1},\ldots ,t_{N}\right)}^{⊤}$ and $y_{n}=p\left(C_{1}|\phi_{n}\right)=\sigma \left(w^{⊤}\phi \right)$. $t_{n}$ represents the label of the data and $y_{n}$ represents the predicted value. We obtain the class of the data by means of integrating the logistic function on the posterior distribution.

We evaluate our methods on eight real-world datasets from the UCI repository [37]: Pima Indian (PI), Haberman (HA), Mammographic (MA), Blood (BL), Cryotherapy (CR), Immunotherapy (IM), Indian (IN), Diabetic (DI) using Bayesian logistic regression. The eight datasets are normalized to have zero mean value and unit variance. We give the Gaussian distribution N(0,100I) as the prior distribution of the parameters.

In each experiment, we run 10,000 iterations with 2000 burn-in samples. We draw leapfrog steps from a uniform distribution Uniform[80,120]. We set step size ϵ=0.00045 and mass matrix m=3I. We use the uniform distribution as the variational distribution. We run each dataset 100 times to calculate the mean and the standard deviation.

Results in terms of the accurate rate of prediction and area under the ROC curve (AUC) [38] are summarized in Table 1 and Table 2. The results show that in these eight datasets, VHMC achieves better performance in classification accuracy rate than in AUC. Compared with other methods, VHMC outperforms HMC and provides a similar performance to VBLR, which indicates that the method proposed in this paper can sample actual posterior distribution.

## 6. Discussion

The most innovative part of our method is that when LHMC refuses to sample, we use the sample generated by the variational distribution to replace the previous sample with a new MH step, thus accelerating the sampling from different modes with a higher acceptance rate. The convergence rate and autocorrelation of LHMC, VHMC and other typical MCMC sampling methods have been shown in Figure 1 and Figure 5. The effectiveness and stability of our method have been demonstrated on both isotropic and heterogeneous Gaussian mixture distributions in Figure 4 and Figure 7. We also compare VHMC with VBLR on the real datasets, where similar performance indicates that our VHMC can successfully sample actual posterior distribution. The object of our method is to approximate target distribution in the probability model, which may not be directly used in other research fields, such as molecular simulation with a more complex system. However, our work can still provide some inspiration for other researchers. It is likely to expand the exploration space and accelerate the target convergence by integrating the variational distribution samples into the MCMC simulation process. For the generation of variational mixture distribution, the traditional machine-learning method uses the EM (Expectation Maximization) algorithm with a slow convergence rate. Here, we adopt the gradient descent method combined with LHMC to estimate the parameters of the variational distribution and demonstrate its effectiveness and stability in different datasets. Of course, more methods are worth further exploration for the effectiveness and robustness of variational distribution in other fields.

## 7. Conclusions

In this study, we propose LHMC to further accelerate the convergence rate and reduce the autocorrelation of samples. We then present VHMC to exploit the information from the variational distribution of the target distribution to make effective distant multi-modal sampling available. A formal theoretical analysis is provided, which demonstrated that VHMC could converge to the target distribution. Our findings are supported by synthetic and real data experiments, which showed that VHMC brings multiple benefits, such as providing superior performance in multi-modal sampling and lower autocorrelation. In the future, we plan to introduce equipotential transformation to the LHMC sampler. If an equipotential sample can be obtained, the autocorrelation of the samples can be significantly reduced.

## Figures and Tables

**Table 1:** Classification accuracies for VBLR, LR, HMC and VHMC on eight data sets.

DATA	LR	VBLR	HMC	VHMC
HA	67.9 ± 0.6	67.7 ± 0.5	67.7 ± 0.4	68.2 ± 0.5
PI	82.5 ± 0.2	82.7 ± 0.2	82.4 ± 0.3	83.1 ± 0.2
MA	89.8 ± 0.1	89.9 ± 0.1	89.9 ± 0.2	89.9 ± 0.2
BL	75.1 ± 0.3	75.3 ± 0.3	71.2 ± 0.6	75.4 ± 0.3
CR	95.4 ± 0.1	95.6 ± 0.1	92.1 ± 0.2	95.7 ± 0.1
IM	77.5 ± 0.4	77.5 ± 0.4	77.4 ± 0.4	77.6 ± 0.4
IN	75.5 ± 0.2	75.8 ± 0.2	75.3 ± 0.3	75.9 ± 0.3
DI	82.6 ± 0.2	82.5 ± 0.2	81.8 ± 0.3	82.5 ± 0.2
RANK	2.75	2.125	3.5	1.25
PVALUE	0.012	0.041	0.056	/

**Table 2:** Area under the ROC curve (AUC) for VBLR, LR, HMC and VHMC on eight data sets.

DATA	LR	VBLR	HMC	VHMC
HA	73.8 ± 0.4	74.6 ± 0.4	74.6 ± 0.2	74.6 ± 0.4
PI	77.0 ± 0.2	77.1 ± 0.2	77.2 ± 0.3	77.6 ± 0.2
MA	82.6 ± 0.2	82.7 ± 0.1	82.5 ± 0.2	82.8 ± 0.1
BL	77.1 ± 0.2	77.1 ± 0.2	74.4 ± 0.5	77.2 ± 0.2
CR	86.9 ± 0.2	87.2 ± 0.2	84.2 ± 0.3	87.3 ± 0.2
IM	84.7 ± 0.2	84.8 ± 0.2	83.8 ± 0.2	84.9 ± 0.2
IN	72.7 ± 0.2	72.8 ± 0.2	71.3 ± 0.3	72.9 ± 0.2
DI	74.4 ± 0.2	74.6 ± 0.2	74.0 ± 0.2	74.7 ± 0.2
RANK	3.125	2.125	3.375	1.125
PVALUE	0.004	0.036	0.018	/

## Data Availability

Not applicable.

## Appendix A. Proof of the Sample Acceptance Rate in VHMC

The acceptance rate $\frac{\pi (x)}{cq(x)}$ of the samples generated from the variational distribution can be justified as follows. The sampling process can be summarized as: X∼q(x), U∼Uniform[0,1] and Y=c·U·q(x), where *c* is a constant. If Y≤π(x), then *x* is accepted. Assume that the probability density function of *x* can be written as:

(A1) $$\begin{array}{c} p_{{}_{X}}\left(x\right)=q\left(x\right), \end{array}$$

where q(x) is a proposal distribution. The probability density function of *y* can be calculated as:

(A2) $$\begin{array}{cc} \; & \;F_{Y}\left(y\right)=P\left(Y\leq y\right)=P\left(c\cdot u\cdot q\left(x\right)\leq y\right)=P\left(u\leq \frac{y}{cq(x)}\right)\\ \; & \;P\left(u\leq \frac{y}{cq(x)}\right)=\int_{0}^{\frac{y}{cq(x)}}du=\frac{y}{cq(x)}\\ \; & \;p_{{}_{Y}}\left(y\right)=F_{Y}^{{}^{\prime }}\left(y\right)={\left(\frac{y}{cq(x)}\right)}^{{}^{\prime }}=\frac{1}{cq(x)}. \end{array}$$

So

(A3) $$\begin{array}{c} P\left(Y\leq \pi \left(x\right)\right)=\frac{\pi (x)}{cq(x)}, \end{array}$$

and the joint probability density of *x* and *y* can be written as:

(A4) $$\begin{array}{c} p\left(x,y\right)=p_{{}_{X}}\left(x\right)\cdot p_{{}_{Y}}\left(y\right)=q\left(x\right)\cdot \frac{1}{cq(x)}=\frac{1}{c}. \end{array}$$

The cumulative distribution function of the accepted samples *d* can be calculated as:

(A5) $$\begin{array}{cc} F(d|accept) & =P\left(X\leq d|Y\leq \pi \left(x\right)\right)=\frac{P(X\leq d,Y\leq \pi (x))}{P(Y\leq \pi (x))}\\ \; & =\frac{\int_{a}^{d}\int_{0}^{\pi (x)}p\left(x,y\right)dxdy}{\pi (x)/cq(x)}\\ \; & =\int_{a}^{d}\pi \left(x\right)dx=F_{{}_{X}}\left(d\right), \end{array}$$

thus, the probability density function of the accepted samples is $F_{{}_{X}}^{{}^{\prime }}\left(d\right)=\pi \left(x\right)$.

## Appendix B. Convergence Proof of VHMC

The correctness of VHMC will be proved in two aspects. First, we prove that π(θ)∝exp(−U(θ)) is the stationary distribution of LHMC dynamics, which consist of Langevin dynamics, Hamiltonian dynamics and Langevin dynamics corresponding to three substages described in Algorithm 2.

For the Langevin dynamics, we assume the stationary distribution of θ obeys $\frac{1}{Z_{\theta }}\exp\left(-f_{1}\left(\theta \right)\right)$ and the transformation probability *T* equals to $\frac{1}{Z_{p}}\exp(-K_{1}\left(p\right)$. If the detailed balance π(i)T(i,j)=π(j)T(j,i) is satisfied, there will exist the transformation probability equation between *i*-th and *j*-th states that

(A6) $$\begin{array}{cc} \frac{1}{Z_{\theta }}\exp\left(-f_{1}\left(\theta_{i}\right)\right)\frac{1}{Z_{p}}\exp\left(-K_{1}\left(p_{i}\right)\right)= & \frac{1}{Z_{\theta }}\exp\left(-f_{1}\left(\theta_{j}\right)\right)\frac{1}{Z_{p}}\exp\left(-K_{1}\left(p_{j}\right)\right),\\ \frac{1}{Z_{\theta }Z_{p}}\exp\left(-f_{1}\left(\theta_{i}\right)-K_{1}\left(p_{i}\right)\right)= & \frac{1}{Z_{\theta }Z_{p}}\exp\left(-f_{1}\left(\theta_{j}\right)-K_{1}\left(p_{j}\right)\right) \end{array}$$

where $K_{1}$ denotes kinetic energy of Langevin dynamics. Since the total energy $H_{1}$ of Langevin dynamics in Equation (12) remains unchanged, we have

(A7) $$\begin{array}{cc} f_{1}\left(\theta \right)+K_{1}\left(p\right)+c= & U_{1}\left(\theta \right)+K_{1}\left(p\right)+Q=H_{1},\\ f_{1}\left(\theta \right)= & U_{1}\left(\theta \right)+c, \end{array}$$

where *c* is a constant. Therefore, the stationary distribution of θ in Langevin dynamics following $\frac{1}{Z_{\theta }}\exp\left(-U_{1}\left(\theta \right)\right)$ is proved.

Similarly, due to the unchanged total energy $H_{2}=U_{2}\left(\theta \right)+K_{2}\left(p\right)$ of Hamiltonian dynamics in Equation (1), we can also obtain the stationary distribution $\frac{1}{Z_{\theta }}\exp\left(-U_{2}\left(\theta \right)\right)$. Now, considering the LHMC, we set the total energy as $H=H_{1}+H_{2}+H_{1}$, which has symmetrical structure. During discrete computation, we can obtain updated θ and *p* by reversible formulations (15)–(17) of Langevin dynamics and (3) of Hamiltonian dynamics. In Algorithm 3, the whole transformation process is reversible under momentum flip due to the symmetrical structure of $H=H_{1}+H_{2}+H_{1}$, which can assure the $\theta_{j}$ falls back to the $\theta_{i}$. If the detailed balance π(i)T(i,j)=π(j)T(j,i) is satisfied, then π is a stationary distribution. In general, the global transformation probability equation can be briefly formulated as follows:

(A8) $$\begin{array}{cc} \; & \exp[H_{1}\left(\theta_{i},p_{i}\right)+H_{2}\left(\left(\theta_{i},p_{i}\right)\right)+H_{1}\left(\left(\theta_{i},p_{i}\right)\right)]=\\ \; & \exp[H_{1}\left(\theta_{j},p_{j}\right)+H_{2}\left(\left(\theta_{j},p_{j}\right)\right)+H_{1}\left(\left(\theta_{j},p_{j}\right)\right)], \end{array}$$

then

(A9) $$\begin{array}{cc} \; & \exp\left(-U_{1}\left(\theta_{i}\right)-U_{2}\left(\theta_{i}\right)-U_{1}\left(\theta_{i}\right)\right)\exp\left(-K_{1}\left(p_{i}\right)-K_{2}\left(p_{i}\right)-K_{1}\left(p_{i}\right)\right)=\\ \; & \exp\left(-U_{1}\left(\theta_{j}\right)-U_{2}\left(\theta_{j}\right)-U_{1}\left(\theta_{j}\right)\right)\exp\left(-K_{1}\left(p_{j}\right)-K_{2}\left(p_{j}\right)-K_{1}\left(p_{j}\right)\right), \end{array}$$

which can prove that $\pi \left(\theta \right)\propto \exp\left(-U_{1}\left(\theta \right)-U_{2}\left(\theta \right)-U_{1}\left(\theta \right)\right)=\exp\left(-U\left(\theta \right)\right)$ is the stationary distribution of the dynamics in LHMC.

Let us consider the detailed balance after introducing the variational distribution: It is clear that in VHMC, the transformation probability $T^{\prime }\left(i,j\right)$ is not a symmetric distribution after sampling from variational distribution and it can be defined as the transformation on target distribution π:

(A10) $$\begin{array}{cc} T^{\prime }\left(i,j\right) & =r\left(i,i_{r}\right)\pi \left(j\right),\\ T^{\prime }\left(j,i\right) & =r\left(j,j_{r}\right)\pi \left(i\right), \end{array}$$

where $r(i,i_{r})$ represents rejection rate of *i* to $i_{r}$, $r(j,j_{r})$ represents rejection rate of *j* to $j_{r}$, and $i_{r}$, $j_{r}$ are the rejected samples. Obviously, the detailed balance is not satisfied. In VHMC, a further MH is introduced to keep the detailed balance. If we introduce the new acceptance rate $r\left(j,j_{r}\right)\pi \left(i\right)$, we can obtain:

(A11) $$\begin{array}{c} \pi \left(i\right)T^{\prime }\left(i,j\right)r\left(j,j_{r}\right)\pi \left(i\right)=\pi \left(j\right)T^{\prime }\left(j,i\right)r\left(i,i_{r}\right)\pi \left(j\right). \end{array}$$

To enlarge the accept rate, we enlarge the whole equation and make the accept rate to be: $\phi =\min\left[1,\frac{\pi \left(j\right)r\left(j,j_{r}\right)\pi \left(i\right)}{\pi \left(i\right)r\left(i,i_{r}\right)\pi \left(j\right)}\right]$. Then the new detailed balance can be written as:

(A12) $$\begin{array}{cc} \pi \left(i\right)T^{\prime }\left(i,j\right)\phi & =\pi \left(i\right)r\left(i,i_{r}\right)\pi \left(j\right)\phi \\ \; & =\min\left[1,\frac{r(j,j_{r})}{r(i,i_{r})}\right]\pi \left(i\right)r\left(i,i_{r}\right)\pi \left(j\right)\\ \; & =\pi \left(j\right)r\left(j,j_{r}\right)\pi \left(i\right)\phi , \end{array}$$

which therefore proves that π is an invariant distribution under the new Metropolis–Hasting criterion.

## References

[1] Sun S., Zhao J. (2020). Pattern Recognition and Machine Learning.

[2] Duane S., Kennedy A.D., Pendleton B.J., Roweth D. (1987). Hybrid Monte Carlo. Phys. Lett. B.

[3] Brooks S., Gelman A., Jones G., Meng X. (2011). Handbook of Markov chain Monte Carlo.

[4] Neal R.M. (2003). Slice sampling. Ann. Stat..

[5] Martino L., Read J. (2013). On the flexibility of the design of multiple try Metropolis schemes. Comput. Stat..

[6] Wang Z., Mohamed S., Freitas N. Adaptive Hamiltonian and Riemann manifold Monte Carlo. Proceedings of the International Conference on Machine Learning.

[7] Hoffman M.D., Gelman A. (2014). The No-U-turn sampler: Adaptively setting path lengths in Hamiltonian Monte Carlo. J. Mach. Learn. Res..

[8] Neal R.M. (1993). Probabilistic Inference Using Markov Chain Monte Carlo Methods.

[9] Celeux G., Hurn M., Robert C.P. (2000). Computational and inferential difficulties with mixture posterior distributions. J. Am. Stat. Assoc..

[10] Neal R.M. (2001). Annealed importance sampling. Stat. Comput..

[11] Rudoy D., Wolfe P.J. Monte Carlo methods for multi-modal distributions. Proceedings of the Asilomar Conference on Signals, Systems and Computers.

[12] Girolami M., Calderhead B. (2011). Riemann manifold Langevin and Hamiltonian Monte Carlo methods. J. R. Stat. Soc. Ser..

[13] Kirkpatrick S., Gelatt C.D., Vecchi M.P. (1983). Optimization by simulated annealing. Science.

[14] Sminchisescu C., Welling M. Generalized darting Monte Carlo. Proceedings of the Artificial Intelligence and Statistics.

[15] Lan S., Streets J., Shahbaba B. Wormhole Hamiltonian Monte Carlo. Proceedings of the Association for the Advancement of Artificial Intelligence.

[16] Tripuraneni N., Rowland M., Ghahramani Z., Turner R. Magnetic Hamiltonian Monte Carlo. Proceedings of the International Conference on Machine Learning.

[17] Swendsen R.H., Wang J.S. (1986). Replica Monte Carlo simulation of spin glasses. Phys. Rev. Lett..

[18] Andricioaei I., Straub J.E., Voter A.F. (2001). Smart darting Monte Carlo. J. Chem. Phys..

[19] Torrie G.M., Valleau J.P. (1977). Nonphysical sampling distributions in Monte Carlo free-energy estimation: Umbrella sampling. J. Comput. Phys..

[20] Blei D.M., Kucukelbir A., McAuliffe J.D. (2017). Variational inference: A review for statisticians. J. Am. Stat. Assoc..

[21] MacKay D.J. (1992). The evidence framework applied to classification networks. Neural Comput..

[22] Gretton A., Borgwardt K.M., Rasch M.J., Schölkopf B., Smola A. (2012). A kernel two-sample test. J. Mach. Learn. Res..

[23] Zhang Y., Wang X., Chen C., Henao R., Fan K., Carin L. Towards unifying Hamiltonian Monte Carlo and slice sampling. Proceedings of the Advances in Neural Information Processing Systems.

[24] Kingma D.P., Ba J. (2014). Adam: A method for stochastic optimization. arXiv.

[25] Leimkuhler B., Reich S. (2004). Simulating Hamiltonian Dynamics.

[26] Brünger A., Brooks III C.L., Karplus M. (1984). Stochastic boundary conditions for molecular dynamics simulations of ST2 water. Chem. Phys. Lett..

[27] Burrage K., Lythe G. (2009). Accurate stationary densities with partitioned numerical methods for stochastic differential equations. SIAM J. Numer. Anal..

[28] Milstein G.N., Tretyakov M.V. (2013). Stochastic Numerics for Mathematical Physics.

[29] Roberts G.O., Stramer O. (2002). Langevin Diffusions and Metropolis-Hastings Algorithms. Methodol. Comput. Appl. Probab..

[30] Kloeden P.E., Platen E. (2013). Numerical Solution of Stochastic Differential Equations.

[31] Leimkuhler B., Matthews C. (2012). Rational construction of stochastic numerical methods for molecular sampling. Appl. Math. Res. Express.

[32] Leimkuhler B., Matthews C. (2013). Robust and efficient configurational molecular sampling via Langevin dynamics. J. Chem. Phys..

[33] Ahn S., Chen Y., Welling M. Distributed and adaptive darting Monte Carlo through regenerations. Proceedings of the Artificial Intelligence and Statistics.

[34] Livingstone S., Betancourt M., Byrne S., Girolami M. (2016). On the geometric ergodicity of Hamiltonian Monte Carlo. arXiv.

[35] Borgwardt K.M., Gretton A., Rasch M.J., Kriegel H.P., Schölkopf B., Smola A.J. (2006). Integrating structured biological data by kernel maximum mean discrepancy. Bioinformatics.

[36] Freedman D.A. (2009). Statistical Models: Theory and Practice.

[37] Asuncion A., Newman D. (2013). UCI Machine Learning Repository.

[38] Hanley J.A., McNeil B.J. (1983). A method of comparing the areas under receiver operating characteristic curves derived from the same cases. Radiology.

